# Associations Between Wearing Masks and Respiratory Viral Infections: A Meta-Analysis and Systematic Review

**DOI:** 10.3389/fpubh.2022.874693

**Published:** 2022-04-27

**Authors:** Yiming Chen, Yuelin Wang, Ningbin Quan, Jun Yang, Yinyin Wu

**Affiliations:** ^1^Department of Epidemiology and Health Statistics, School of Public Health, Faculty of Medicine, Hangzhou Normal University, Hangzhou, China; ^2^Department of Nutrition and Toxicology, School of Public Health, Faculty of Medicine, Hangzhou Normal University, Hangzhou, China; ^3^National Key Laboratory for the Diagnosis and Treatment for Infectious Diseases, The First Affiliated Hospital, Zhejiang University School of Medicine, Hangzhou, China

**Keywords:** masks, effectiveness, respiratory viral infections, meta-analysis, systematic review

## Abstract

**Background:**

Respiratory viral infections (RVIs) are a major health concern, and some previous studies have shown that wearing masks was effective in preventing RVIs, while others failed to show such effect. Therefore, a systematic review and meta-analysis was conducted to investigate the effectiveness of wearing masks.

**Methods:**

PubMed, ScienceDirect, Web of Science, the Cochrane Library, EMBASE, MEDLINE, China National Knowledge Infrastructure (CNKI), and Chinese Scientific Journal Database (VIP database) were searched for studies evaluating the effectiveness of wearing masks. The risk ratio (RR) was used to measure the effectiveness of wearing masks in preventing RVIs for randomized controlled trials (RCTs) and cohort studies, and the odds ratio (OR) was used for case-control studies. Forest plots were used to visually assess pooled estimates and corresponding 95% CIs. The *I*^2^ test was used to examine the heterogeneity, and subgroup analysis was used to explore the possible explanations for heterogeneity or compare the results between subgroups. Sensitivity analysis was conducted to assess robustness of the synthesized results. Begg's test and Egger's test were used to assess the publications bias.

**Results:**

Thirty-one studies (13,329 participants) were eligible for meta-analyses. Overall, the results showed that wearing masks was effective in preventing RVIs. The sensitivity analysis showed that the results of those meta-analyses were robust and reliable. There was no significant publication bias in meta-analysis of case-control studies and most subgroup analyses.

**Conclusions:**

Wearing masks might be effective in preventing RVIs. To reduce their RVI risk, people should wear masks when they go out in public.

**Systematic Review Registration:**

https://www.crd.york.ac.uk/PROSPERO/, identifier: CRD42021296092.

## Introduction

In recent years, respiratory viral infections (RVIs), such as Corona Virus Disease 2019 (COVID-19), Severe Acute Respiratory Syndrome (SARS), influenza, and Middle East Respiratory Syndrome (MERS), have spread across the world and seriously threatened public health. Under such circumstances, there is an urgent need to find some effective management strategies that can help prevent RVIs. Previous studies have found that surgical masks and N95 masks were effective in preventing RVIs (1–4), as were common masks, such as cotton masks (5, 6). Thus, in the combat against COVID-19, people were required to wear masks when going out in public in many countries (7–9). However, some studies indicated that there was insufficient evidence for the effectiveness of wearing masks (10, 11), while substantial adverse physiological and psychological effects of wearing masks, including hypercapnia, shortness of breath, anxiety, depression, etc. (12), were reported. Several meta-analyses have evaluated the potential benefits of wearing masks, however, they all suffered certain weakness, for instance, some only analyzed a single disease (13–15), some focused on limited types of masks (16–20), and others only included a small number of studies (13, 21). Moreover, the conclusions of these meta-analyses were inconsistent, as some found that wearing masks were effective in preventing RVIs (13–16, 18, 21), while another study failed to show the benefits (17, 19, 20). In view of this problem, a meta-analysis was conducted to quantify the effectiveness of wearing masks in the prevention of RVIs.

## Materials and Methods

A systematic review was conducted following PRISMA guidelines (22). The study protocol has been registered with PROSPERO: CRD42021296092.

### Search Strategy

A comprehensive literature search was carried out in PubMed, ScienceDirect, Web of Science, the Cochrane Library, EMBASE, MEDLINE, China National Knowledge Infrastructure (CNKI), and Chinese Scientific Journal Database (VIP database) from January 1, 2000 to May 1, 2021. The literature search was conducted using the following medical subject heading terms and Boolean operators: “(“mask” OR “facemask” OR “N95” OR “respirator”) AND (“influenza virus” OR “SARS” OR “MERS” OR “COVID-19” OR “virus”).” The details of the search strategy are shown in Supplementary Table 1. Searching was restricted to articles in English and Chinese, and the references of the articles retrieved were also screened.

### Inclusion and Exclusion Criteria

Inclusion criteria were (1) study type: case-control studies, cohort studies, and randomized controlled trials (RCTs); (2) participants: healthcare workers (HCWs, workers in a health care setting who might be exposed to patients with RVIs) and non-healthcare workers (non-HCWs); (3) intervention: all types of masks; and (4) outcome: laboratory-confirmed RVIs. Exclusion criteria were (1) studies without raw data, such as theoretical models, conference abstracts, case reports, editorials, and comments; (2) studies with incomplete or invalid data; (3) studies with unavailable full texts; (4) human or non-human experimental laboratory studies; and (5) duplicate publication or overlapped studies.

### Study Selection and Data Extraction

Two reviewers independently screened the articles based on the titles, abstracts, and full texts. Then, two reviewers independently exacted the following data from the included studies: first author, publication year, country, type of RVI, type of mask, occupation of participants, sample size, and study design. Any disagreements were resolved by a panel discussion with other reviewers.

### Quality Assessment

The Newcastle-Ottawa Scale (NOS) (23, 24) was used to evaluate the quality of the case-control studies and cohort studies. The scale, whose ratings ranged from zero to nine, included eight items within three domains to evaluate bias in selection, comparability, and exposure (for case-control studies)/outcome (for cohort studies). A scale of six to nine represented high quality, and scale of five or less represented low quality of the study. The Cochrane Collaboration's tool (25) was used for evaluating the quality of RCTs. The tool covers six domains of bias: selection bias, performance bias, detection bias, attrition bias, reporting bias, and other bias. Each domain was assessed as low, unclear or high risk of bias. Two reviewers completed assessments independently, and any disagreements were resolved by a panel discussion with other reviewers.

### Statistical Analysis

Data analysis was performed by using the Review Manager 5.3 software and STATA 14.0 software. The risk ratio (RR) was used to measure the effectiveness of wearing masks in preventing RVIs for RCTs and cohort studies, and the odds ratio (OR) was used for case-control studies. Forest plots were used to visually assess pooled estimates and corresponding 95% CIs. The heterogeneity was examined by the *I*^2^ test. A random-effects model was used to calculate the pooled effect size when the heterogeneity was considered significant (*I*^2^ > 50%, *P* ≤ 0.10); otherwise, a fixed-effects model was used. Subgroup analysis was used to explore the possible explanations for heterogeneity or compare the results between subgroups. Leave-one-out sensitivity analysis was conducted to assess robustness of the synthesized results. Begg's test and Egger's test were used to assess the publication bias, and *P* < 0.05 was set as the level of significance.

## Results

### Literature Search

**Figure 1:**
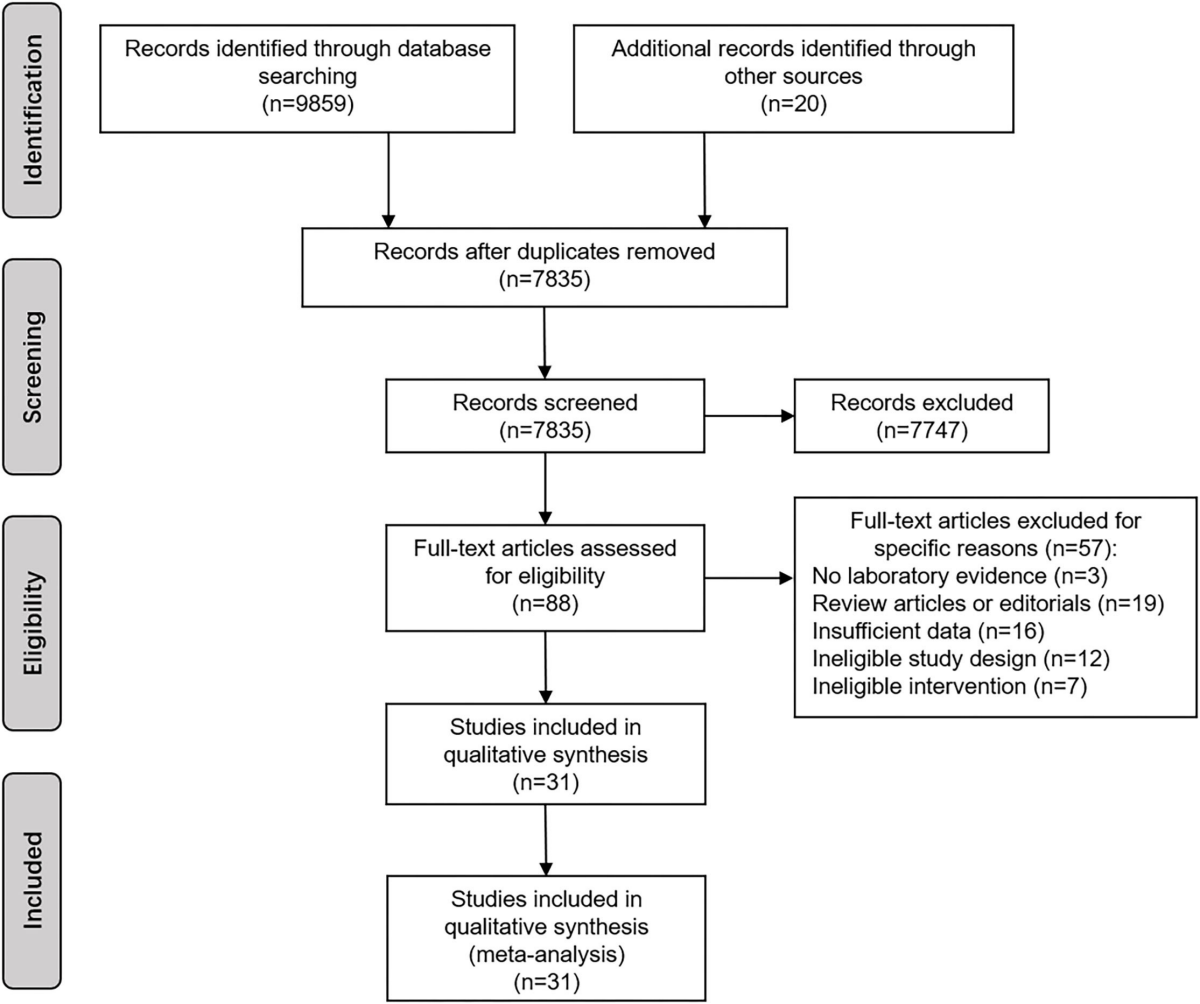
The study selection process.

After searching the databases, 9,859 articles were identified, and finally 31 articles (1–6, 10, 11, 26–48) were included in the final pooled analysis based on the inclusion/exclusion criteria, and the total number of participants involved in the systematic review was 13,329. The search details of the study selection process are shown in Figure 1, and a summary of the included studies are presented in Table 1. Among them, 18 articles (2, 5, 6, 29–32, 34, 36, 38, 40–48) were case-control studies, 7 articles (1, 4, 26, 28, 33, 39, 45) were cohort studies, and 6 articles (3, 10, 11, 27, 35, 37) were RCTs. In case-control studies, 14 studies (2, 5, 6, 29–31, 34, 36, 38, 43, 44, 46–48) were of high quality (Supplementary Table 2). In cohort studies, 3 studies (1, 28, 33) were of high quality (Supplementary Table 3). In RCTs, the results of The Cochrane Collaboration's tool present an overall low risk of bias (Supplementary Figures 1, 2).

**Table 1:** Characteristics of studies included in the meta-analysis.

**References**	**Country**	**Virus**	**Method or index used for confirming the cases**	**Mask type**	**Occupation of participants**	**Sample size of case (experimental) group/control group**	Study quality^*^
**Case-control studies**							
Chokephaibulkit et al. (29)	Thailand	H1N1	HI titer ≥ 40	Masks not defined	HCWs	33/223	7 (high)
Doung-Ngern et al. (30)	Thailand	SARS-CoV-2	RT-PCR	Masks not defined	Non-HCWs	131/698	9 (high)
Guo et al. (31)	China	SARS-CoV-2	RT-PCR	N95 masks	HCWs	24/48	7 (high)
Heinzerling et al. (32)	United States	SARS-CoV-2	RT-PCR	Surgical masks	HCWs	3/34	5 (low)
Khalil et al. (34)	Bangladesh	SARS-CoV-2	RT-PCR	N95 masks	HCWs	98/92	7 (high)
Ki et al. (2)	Korea	MERS-CoV	RT-PCR	Masks not defined	HCWs	6/442	6 (high)
Ma et al. (36)	China	SARS-CoV	RT-PCR/ELISA	Masks not defined	HCWs	239/180	7 (high)
Nishiura et al. (38)	Vietnam	SARS-CoV	ELISA	Surgical masks	HCWs and non-HCWs	29/116	6 (high)
Pei et al. (5)	China	SARS-CoV	IgG-antibody was positive	Common masks	HCWs	133/281	8 (high)
Reynolds et al. (40)	Vietnam	SARS-CoV	RT-PCR	Masks not defined	HCWs	22/45	4 (low)
Scales et al. (41)	Canada	SARS-CoV	PCR	Masks not defined	HCWs	7/24	5 (low)
Seto et al. (42)	China	SARS-CoV	IIFA	Masks not defined	HCWs	13/241	4 (low)
Teleman et al. (43)	Singapore	SARS-CoV	Serological identification	N95 masks	HCWs	36/50	7 (high)
Tuan et al. (44)	Vietnam	SARS-CoV	RT-PCR/ELISA	Masks not defined	Non-HCWs	7/156	6 (high)
Wu et al. (46)	China	SARS-CoV	ELISA	Masks not defined	Non-HCWs	94/281	8 (high)
Yin et al. (6)	China	SARS-CoV	RT-PCR/ELISA	Common masks	HCWs	77/180	7 (high)
Zhang et al. (48)	China	H1N1	RT-PCR	Masks not defined	HCWs	51/204	7 (high)
Zhang et al. (47)	China	SARS-CoV-2	RT-PCR/ELISA	Masks not defined	Non-HCWs	14/14	6 (high)
**Cohort studies**							
Alraddadi et al. (26)	Saudi Arabia	MERS-CoV	RT-PCR	Masks not defined	HCWs	284/98	5 (low)
Cheng et al. (28)	China	H1N1	RT-PCR	Surgical masks	Non-HCWs	538/268	7 (high)
Jaeger et al. (33)	Korea	H1N1	HI	Masks not defined	HCWs	20/43	7 (high)
Loeb et al. (1)	Canada	SARS-CoV	IFA	Masks not defined	HCWs	23/9	7 (high)
Nishiyama et al. (39)	Vietnam	SARS-CoV	ELISA	Masks not defined	HCWs	61/18	5 (low)
Wang et al. (4)	China	SARS-CoV-2	Molecular diagnosis	N95 masks	HCWs	278/213	5 (low)
Wang et al. (45)	China	SARS-CoV-2	RT-PCR/ gene sequencing	Masks not defined	Non-HCWs	46/41	5 (low)
**RCTs**							
Ailello et al. (11)	United States	Influenza virus not defined	RT-PCR	Masks not defined	Non-HCWs	392/370	-
Bundgaard et al. (27)	Denmark	SARS-CoV-2	RT-PCR	Surgical masks	Non-HCWs	2392/2470	-
Cowling et al. (10)	China	H5N1	PCR	Surgical masks	Non-HCWs	29/95	-
Larson et al. (35)	United States	Influenza virus not defined	PCR	Surgical masks	Non-HCWs	50/48	-
MacIntyre et al. (37)	Vietnam	Respiratory viruses not defined	RT-PCR	Masks not defined	HCWs	580/458	-
Suess et al. (3)	Germany	Influenza virus not defined	RT-PCR	Surgical masks	Non-HCWs	69/82	-

*MERS-CoV, Middle East Respiratory Syndrome Coronavirus; SARS-CoV-2, Severe Acute Respiratory Syndrome Coronavirus 2; H1N1, Influenza A Virus, H1N1 Subtype; H5N1, Influenza A Virus, H5N1 Subtype; SARS-CoV, Severe Acute Respiratory Syndrome Coronavirus; HCWs, healthcare workers; non-HCWs, non-healthcare workers; RT-PCR, reverse transcriptase-polymerase chain reaction; HI, hemagglutination inhibition; ELISA, enzyme linked immunosorbent assay; IIFA, indirect immunofluorescence assay; IFA, immunofluorescence assay; PCR, polymerase chain reaction; RCTs, randomized controlled trials*;

* *The ratings of Newcastle-Ottawa Scale for case-control studies and cohort studies*.

### Effectiveness of Wearing Masks in Preventing RVIs

Three meta-analyses were conducted according to the type of study design.

**Figure 2:**
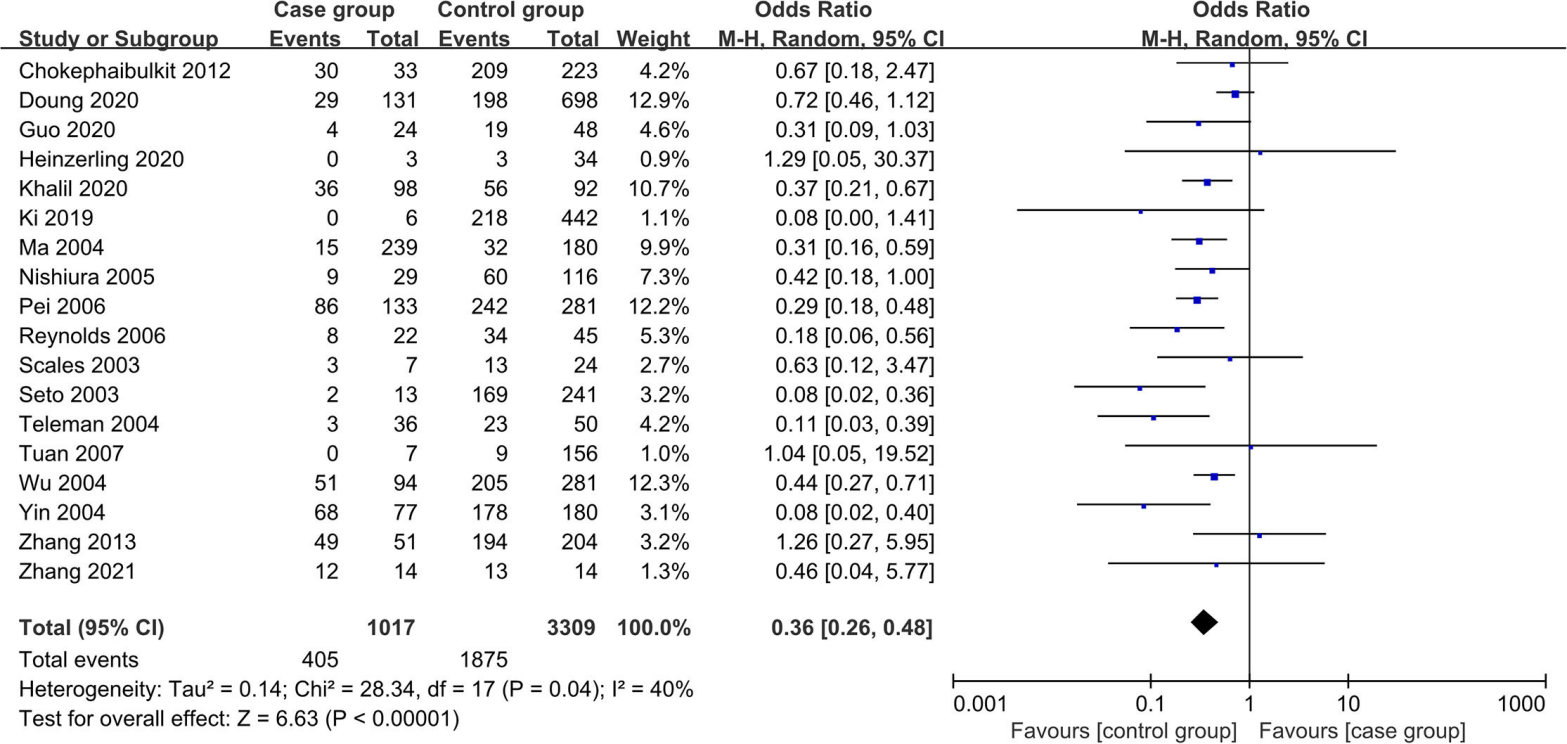
Forest plot of meta-analysis of case-control studies.

In the meta-analysis of case-control studies, 18 studies were included, and the total number of participants was 4,326. The *I*^2^ test indicated significant heterogeneity among the studies (*I*^2^ = 40.00%, *P* = 0.04), so a random-effects model was used to pool the data. The result suggested that wearing masks was effective in preventing RVIs (OR = 0.36, 95% CI: 0.26~0.48, *P* < 0.01; see Figure 2).

**Figure 3:**
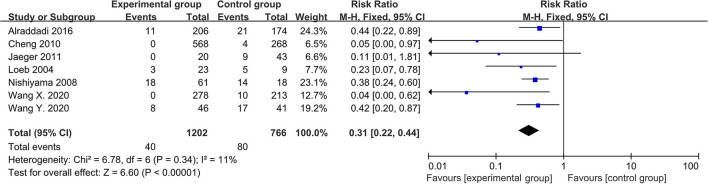
Forest plot of meta-analysis of cohort studies.

In the meta-analysis of cohort studies, 7 studies were included, and the total number of participants was 1,968. The *I*^2^ test indicated no significant heterogeneity among the studies (*I*^2^ = 11.00%, *P* = 0.34), so a fixed-effects model was used to pool the data. The result suggested that wearing masks was effective in preventing RVIs (RR = 0.31, 95% CI: 0.22~0.44, *P* < 0.01; see Figure 3).

**Figure 4:**
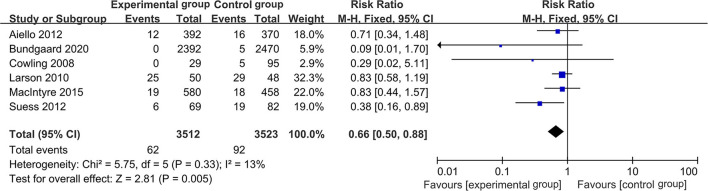
Forest plot of meta-analysis of RCTs.

In the meta-analysis of RCTs, 6 studies were included, and the total number of participants was 7,035. The *I*^2^ test indicated no significant heterogeneity among the studies (*I*^2^ = 13.00%, *P* = 0.33), so a fixed-effects model was used to pool the data. The result suggested that wearing masks was effective in preventing RVIs (RR = 0.66, 95% CI: 0.50~0.88, *P* = 0.01; see Figure 4).

### Subgroup Analyses

Three subgroup analyses based on type of RVI, type of mask, and occupation of participants were conducted respectively for every meta-analysis (Table 2).

**Table 2:** The results of meta-analyses.

**Category**	**Subgroup**	**N^a^**	**OR/RR (95%CI)**	**P^b^**	**Test of heterogeneity**		***P*** **-value of publication bias assessment** ^c^	
					***P-*value**	***I^**2**^*(%)**	**Begg's test**	**Egger's test**
Case-control studies		18	0.36 (0.26~0.48)	<0.01	0.04	40.00	>0.99	0.31
RVI	SARS	10	0.28 (0.20~0.41)	<0.01	0.16	31.40	0.47	0.24
	MERS	1	0.08 (0.004~1.41)	0.08	-	-	-	-
	H1N1	2	0.87 (0.32~2.36)	0.79	0.54	<0.01	-	-
	COVID-19	5	0.53 (0.37~0.77)	<0.01	0.37	5.80	0.81	0.74
Mask	N95 masks	3	0.27 (0.14~0.54)	<0.01	0.23	32.70	-	-
	Surgical masks	2	0.45 (0.20~1.05)	0.06	0.50	<0.01	-	-
	Common masks	2	0.20 (0.06~0.62)	<0.01	0.13	55.50	-	-
	Masks not defined	11	0.42 (0.28~0.64)	<0.01	0.07	41.10	0.88	0.42
Occupation	HCWs	12	0.29 (0.20~0.42)	<0.01	0.16	29.40	0.84	0.92
	Non-HCWs	5	0.56 (0.40~0.78)	<0.01	0.39	3.30	0.81	0.57
	HCWs and non-HCWs	1	0.42 (0.18~1.00)	0.05	-	-	-	-
Cohort studies		7	0.31 (0.22~0.44)	<0.01	0.34	11.00	0.04	0.01
RVI	SARS	2	0.34 (0.22~0.53)	<0.01	0.45	<0.01	-	-
	MERS	1	0.44 (0.22~0.89)	0.02	-	-	-	-
	H1N1	2	0.08 (0.01~0.61)	0.01	0.72	<0.01	-	-
	COVID-19	2	0.27 (0.13~0.53)	<0.01	0.07	70.40	-	-
Mask	N95 masks	2	0.30 (0.16~0.58)	<0.01	0.07	69.30	-	-
	Surgical masks	1	0.05 (0.00~0.97)	<0.05	-	-	-	-
	Masks not defined	4	0.34 (0.23~0.51)	<0.01	0.68	<0.01	-	-
Occupation	HCWs	5	0.30 (0.20~0.45)	<0.01	0.30	17.80	0.09	0.048
	Non-HCWs	2	0.33 (0.16~0.65)	<0.01	0.16	49.00	-	-
RCTs		6	0.66 (0.50~0.88)	0.01	0.33	13.00	0.06	0.048
RVI	Influenza not defined	3	0.67 (0.49~0.93)	0.02	0.22	34.70	-	-
	H5N1	1	0.29 (0.02~5.11)	0.40	-	-	-	-
	COVID-19	1	0.09 (0.01~1.70)	0.11	-	-	-	-
	RVIs not defined	1	0.83 (0.44~1.57)	0.57	-	-	-	-
Mask	Surgical masks	5	0.65 (0.48~0.89)	0.01	0.21	31.80	0.22	0.09
	Masks not defined	1	0.71 (0.34~1.48)	0.36	-	-	-	-
Occupation	HCWs	1	0.83 (0.44~1.57)	0.57	-	-	-	-
	Non-HCWs	5	0.62 (0.45~0.85)	<0.01	0.21	32.30	0.22	0.06

*RVI, respiratory virus; SARS, Severe Acute Respiratory Syndrome; MERS, Middle East Respiratory Syndrome; H1N1, influenza A (H1N1); COVID-19, Corona Virus Disease 2019; H5N1, influenza A (H5N1); HCWs, healthcare workers; non-HCWs, non-healthcare workers; RCTs, randomized controlled trials*;

a *Number of studies*;

b *P value for OR/RR*;

c *Publication bias assessment was conducted when the total number of studies was equal or >5*.

#### Subgroup Analyses of Case-Control Studies

In the subgroup analysis based on type of RVI, the *I*^2^ test indicated no significant heterogeneity in every subgroup. The result showed that masks were effective in preventing SARS (OR = 0.28, 95% CI: 0.20~0.41) and COVID-19 (OR = 0.53, 95% CI: 0.37~0.77), while there was no significant effectiveness of wearing masks in preventing MERS (OR = 0.08, 95% CI: 0.004~1.41) and H1N1 (OR = 0.87, 95% CI: 0.32~2.36).

In the subgroup analysis based on type of mask, the *I*^2^ test indicated significant heterogeneity in the subgroup of common masks (*I*^2^ = 55.50%, *P* = 0.13) and masks not defined (*I*^2^ = 40.10%, *P* = 0.07). The result showed that N95 masks (OR = 0.27, 95% CI: 0.14~0.54) and common masks (OR = 0.20, 95% CI: 0.06~0.62) were both effective in preventing RVIs, while surgical masks (OR = 0.45, 95% CI: 0.20~1.05) failed to show the significant effectiveness.

In the subgroup analysis based on occupation of participants, the *I*^2^ test indicated no significant heterogeneity in each subgroup. The result showed significant effectiveness of wearing masks in preventing RVIs for both HCWs (OR = 0.29, 95% CI: 0.20~0.42) and non-HCWs (OR = 0.56, 95% CI: 0.40~0.78).

#### Subgroup Analyses of Cohort Studies

In the subgroup analysis based on type of RVI, the *I*^2^ test indicated significant heterogeneity in the subgroup of COVID-19 (*I*^2^ = 70.40%, *P* = 0.07). The result showed that masks were effective in preventing SARS (RR = 0.34, 95% CI: 0.22~0.53), MERS (RR = 0.44, 95% CI: 0.22~0.89), H1N1 (RR = 0.08, 95% CI: 0.01~0.61), and COVID-19 (RR = 0.27, 95% CI: 0.13~0.53).

In the subgroup analysis based on type of mask, the *I*^2^ test indicated significant heterogeneity in the subgroup of N95 masks (*I*^2^ = 69.30%, *P* = 0.07). The result showed that N95 masks (RR = 0.30, 95% CI: 0.16~0.58) and surgical masks (RR = 0.05, 95% CI: 0.00~0.97) were all effective in preventing RVIs.

In the subgroup analysis based on occupation of participants, the *I*^2^ test indicated no significant heterogeneity in each subgroup. The result showed significant effectiveness of wearing masks in preventing RVIs for both HCWs (RR = 0.30, 95% CI: 0.20~0.45) and non-HCWs (RR = 0.33, 95% CI: 0.16~0.65).

#### Subgroup Analyses of RCTs

In the subgroup analysis based on type of RVI, the *I*^2^ test indicated no significant heterogeneity in the subgroup of influenza not defined (*I*^2^ = 34.70%, *P* = 0.22). The result showed that masks were effective in preventing influenza (RR = 0.67, 95% CI: 0.49~0.93), while there was no significant effectiveness showed in other subgroups.

In the subgroup analysis based on type of mask, the *I*^2^ test indicated no significant heterogeneity in the subgroup of surgical masks (*I*^2^ = 31.80%, *P* = 0.21). The result showed that surgical masks (RR = 0.65, 95% CI: 0.48~0.89) were effective in preventing RVIs.

In the subgroup analysis based on occupation of participants, the *I*^2^ test indicated no significant heterogeneity in the subgroup of non-HCWs (*I*^2^ = 32.30%, *P* = 0.21). The result showed significant effectiveness of wearing masks in preventing RVIs for non-HCWs (RR = 0.62, 95% CI: 0.45~0.85).

### Sensitivity Analysis and Publication Bias

The sensitivity analysis showed that the results of meta-analyses including case-control studies (Supplementary Figure 3), cohort studies (Supplementary Figure 4), and RCTs (Supplementary Figure 5) were all robust and reliable.

There was no significant publication bias in the meta-analysis of case-control studies, while the meta-analyses of cohort studies and RCTs were of significant publication biases. However, most subgroup analyses showed no significant publication bias (Table 2).

## Discussion

In this meta-analysis, the associations between wearing masks and the risk of RVIs were analyzed, and the results showed that wearing masks can reduce the risk of RVIs overall.

In previous meta-analyses, Liang et al. (21) and Offeddu et al. (16) investigated the effectiveness of wearing masks in the prevention of RVIs, and both results showed that wearing masks could significantly reduce the risk of RVIs. The results of this study were consistent with these results. For specific type of RVIs, Li et al. (14), Chu et al. (13), and Tabatabaeizadeh et al. (15) found that mask use provided a significant effectiveness in preventing COVID-19, while Sharma et al. (17) failed to find the effectiveness.

The major transmission routes of respiratory viruses are inhalation of aerosol (≤5 μm)/droplet (>5 μm) and person-to-person contact. Aerosol/droplets with respiratory viruses can transmit to susceptible individuals when patients with RVIs are speaking, coughing, or sneezing (49–51). Masks that can filtrate aerosol/droplets provide susceptible individuals with physical protection against respiratory viruses, thus reducing the risk of RVIs. A study examining the filtration efficiency of masks for polystyrene latex microspheres sized from 0.03~2.5 μm showed that the filtration efficiency of surgical masks was 76~92%, that of N95 masks was 76~92%, and that of cloth masks with an exhaust valve was 39~65% (52). Whiley et al. (53) found that the filtration efficiency of surgical masks, N95 masks, and three-layered cotton masks was 99.3, 98.5, and 65.8%, respectively, when the size of microspheres was 2.6 μm; and that the filtration efficiency became 99.9, 99.6, and 54.4%, respectively, when the size of the microspheres was 6 μm. Patra et al. (54) examined the efficiency of some commonly used face masks in filtrating microspheres sized from 0.3~10 μm, and found out that the filtration efficiency of N95 masks, which proved to be the most effective, was 91.8%; the filtration efficiency of surgical masks was 77.8%, and the filtration efficiency of one-layered T-shirt fabric masks was 64.8% and the least effective. Nonetheless, these studies showed that masks can filtrate aerosol/droplets.

For the subgroup analyses based on type of RVI, the result showed no significant effectiveness of masks in preventing H1N1 and MERS in case-control studies, while the subgroup analysis of cohort studies showed opposite results. Moreover, the result of the subgroup analysis of RCTs showed no significant effectiveness of masks in preventing H5N1. Given that the total number of studies investigating H1N1, MERS, or H5N1 was inadequate, more studies should be conducted to make the evidence stronger. For the subgroup analyses based on type of mask, the result showed no significant effectiveness of surgical masks in case-control studies, the reason also might be that the total number of studies in the subgroup was inadequate. In contrast, there were 5 RCTs investigating the effectiveness of surgical masks, and the result showed significant effectiveness when the data of these 5 RCTs were pooled (The publication bias was not significant). Thus, it could be considered that surgical masks were effective in preventing RVIs. Based on the results of the subgroup analyses for participants occupation, it could be considered that masks were effective for both HCWs and non-HCWs.

### Study Limitations

The study has some limitations. First, besides wearing masks, some participants might take other measures to prevent RVIs, such as hand hygiene, and wearing gloves/goggles/full face shields. But this information was few available. Thus, the potential impacts of these factors on the outcome could not be considered. Also, the possible influence of location and contact distance was not be analyzed. Second, in different region, the epidemic types and strength of RVIs, as well as people's living environments and habits, might be different. Unfortunately, no studies from Africa, South America, or Oceania were included in this meta-analysis, so the effectiveness of wearing masks in these areas was unknown. Moreover, the total number of studies was inadequate in some subgroups, more studies should to be conducted to make the evidence stronger. Finally, there was significant publication biases in the meta-analyses of cohort studies and RCTs. The reason might be that the number of high-quality studies was relatively inadequate.

### Conclusions

Overall, wearing masks was effective in preventing RVIs, especially SARS, influenza, and COVID-19. Besides, N95 masks, surgical masks, and common masks were all effective for RVIs prevention. This suggests that people should be encouraged to wear masks when they are in a large group of people to reduce the risk of RVIs. And such Infection Prevention and Control (IPC) strategies are recommended to be implemented to mitigate the RVIs rates.

## Data Availability Statement

The original contributions presented in the study are included in the article/Supplementary Material, further inquiries can be directed to the corresponding authors.

## Author Contributions

YWu and JY designed the study and revised the manuscript critically for important intellectual content. YWa, NQ, and YC conducted the systematic literature search and data extraction. YC conducted the statistical analyses and wrote the manuscript. All authors contributed to the article and approved the submitted version.

## Funding

This work was supported by National Natural Science Foundation of China [31971138]; Natural Science Foundation of Zhejiang Province [LZ19H260001]; and Health Commission of Zhejiang Province [2022506699].

## Conflict of Interest

The authors declare that the research was conducted in the absence of any commercial or financial relationships that could be construed as a potential conflict of interest.

## Publisher's Note

All claims expressed in this article are solely those of the authors and do not necessarily represent those of their affiliated organizations, or those of the publisher, the editors and the reviewers. Any product that may be evaluated in this article, or claim that may be made by its manufacturer, is not guaranteed or endorsed by the publisher.
