# Supplementary material for: Associations Between Wearing Masks and Respiratory Viral Infections: A Meta-Analysis and Systematic Review
Source: Front Public Health. 2022 Apr 27;10:874693. doi: 10.3389/fpubh.2022.874693 (PMC9092448; doi:10.3389/fpubh.2022.874693)
Supplement: Supplementary file 1 [file Data_Sheet_1.DOCX]

Supplementary Material

# Supplementary Figures and Tables

## Supplementary Figures


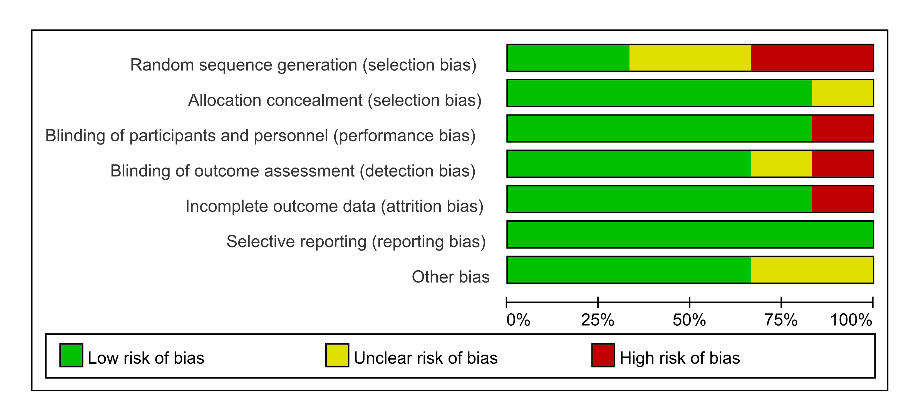


**Supplementary Figure 1.** Risk of bias graph in RCTs

**
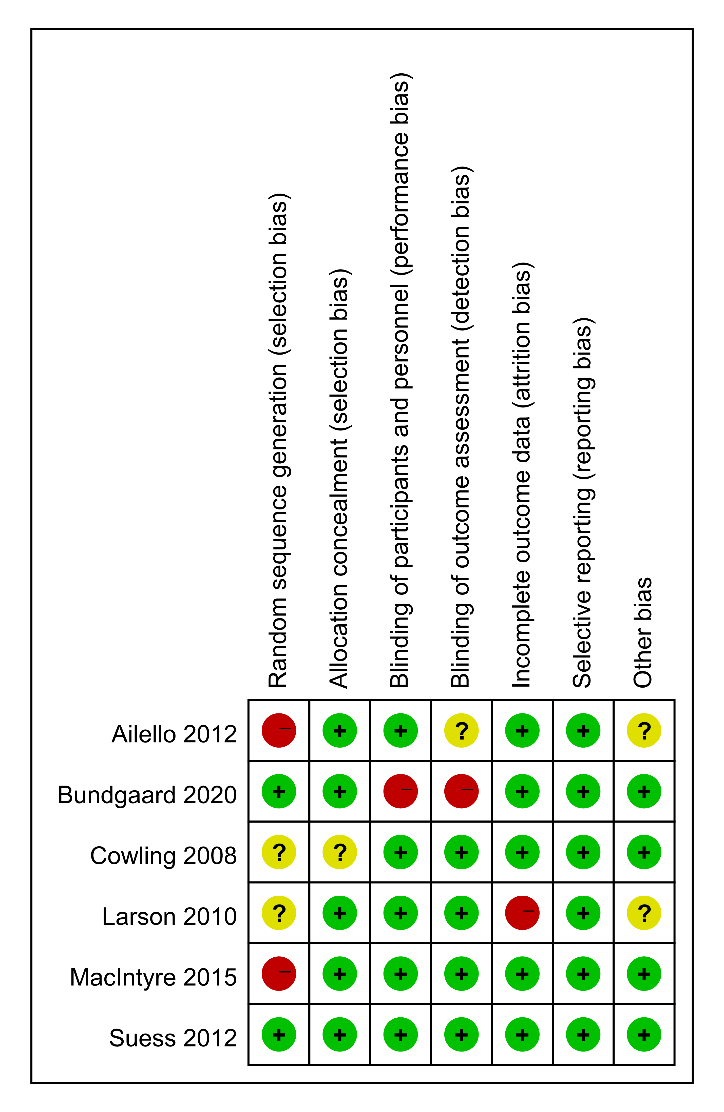
**

**Supplementary Figure 2.** Risk of bias summary in RCTs

**
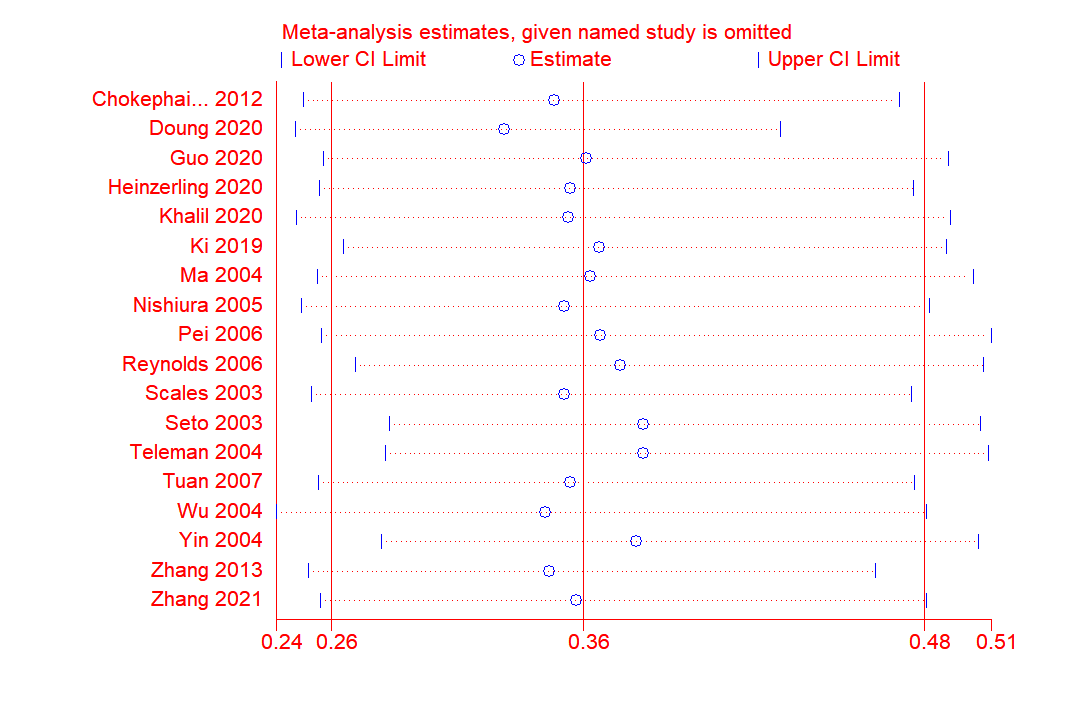
**

**Supplementary Figure 3.** Sensitivity analysis of case-control studies

**
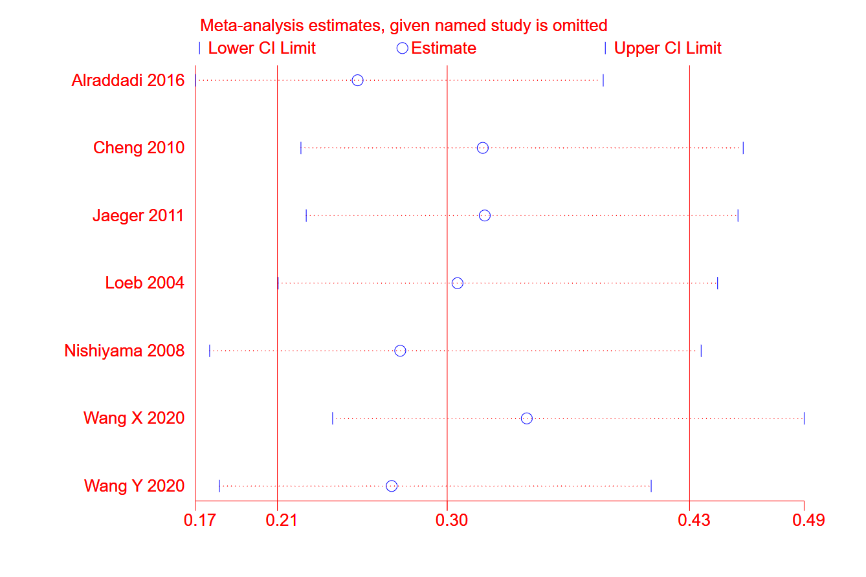
**

**Supplementary Figure 4.** Sensitivity analysis of cohort studies

**
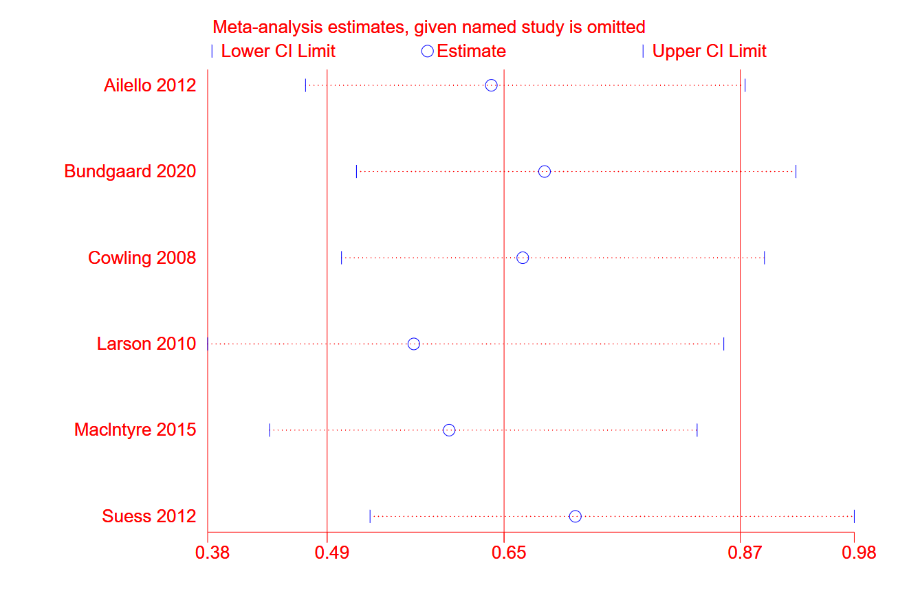
**

**Supplementary Figure 5.** Sensitivity analysis of RCTs

## Supplementary Tables

**Supplementary Table 1.** The details of the search strategy

| Database | Detailed Search Strings/MeSH Terms | |
| --- | --- | --- |
| PubMed (NCBI) | (("masks"[MeSH Terms] OR "masks"[Text Word] OR "facemask"[Text Word] OR "N95"[Text Word] OR "respirator"[Text Word] OR "ventilators"[Text Word]) AND ("influenza a virus"[MeSH Terms] OR "influenza a virus"[Text Word] OR ("influenza b virus"[MeSH Terms] OR ("influenza b virus"[MeSH Terms] OR "influenza b virus"[All Fields]) OR "coronavirus infections"[MeSH Terms] OR "severe acute respiratory syndrome coronavirus 2"[Text Word] OR "SARS-CoV-2"[Text Word] OR "COVID-19"[Text Word] OR "2019-nCoV"[Text Word] OR ("SARS"[All Fields] AND "infections"[MeSH Terms]) OR (("coronavirus infections"[MeSH Terms] OR ("coronavirus"[All Fields] AND "infections"[All Fields]) OR "coronavirus infections"[All Fields] OR "mers"[All Fields]) AND "infections"[MeSH Terms]) OR "middle east respiratory syndrome"[Text Word]))) | |
| Web of Science | TS=("mask" OR "face mask" OR "facemask" OR "N95" OR "*mask" OR "respirator") AND TS=("influenza A virus" OR "influenza B virus" OR "respiratory syncytial virus" OR "parainfluenza virus" OR "adenovirus" OR" SARS" OR "MERS" OR "COVID-19" OR *virus) | |
| The Cochrane Library | mask in Title Abstract Keyword AND virus in Title Abstract Keyword - (Word variations have been searched) | |
| Chinese National Knowledge Infrastructure (CNKI) | ( (主题=口罩 或者 题名=口罩 或者 v_subject=中英文扩展(口罩,中英文对照) 或者 title=中英文扩展(口罩,中英文对照)) 并且 (主题=病毒 或者 题名=病毒 或者 v_subject=中英文扩展(病毒,中英文对照) 或者 title=中英文扩展(病毒,中英文对照)) ) (模糊匹配),专辑导航：全部; 数据库：文献 跨库检索 | |
| VIP (Chinese) database | 文摘=口罩 AND 文摘=病毒 | |
| Science Direct | ("mask" OR "facemask" OR "N95" OR "respirator") AND　("influenza virus" OR" SARS" OR "MERS" OR "COVID-19" OR "virus"） | |
| Embase and Medline | #1 | 'mask'/exp OR 'mask' OR 'facemask'/exp OR 'facemask' OR 'n95'/exp OR 'n95' OR 'respirator'/exp OR 'respirator' |
|  | #2 | (influenza OR sars OR mers OR 'covid 19' OR virus) |
|  | #3 | #1 AND #2 AND ([controlled clinical trial]/lim OR [randomized controlled trial]/lim) AND [article]/lim AND [humans]/lim AND ([embase]/lim OR [medline]/lim) |

**Supplementary Table 2.** The quality of the case-control studies

| First author, year | Selection | Comparability | Exposure | The ratings of Newcastle-Ottawa Scale |
| --- | --- | --- | --- | --- |
| Chokephaibulkit,2012 | 3 | 2 | 2 | 7 |
| Doung,2020 | 4 | 2 | 3 | 9 |
| Guo,2020 | 3 | 2 | 2 | 7 |
| Heinzerling,2020 | 3 | 1 | 1 | 5 |
| Khalil,2020 | 3 | 2 | 2 | 7 |
| Ki,2019 | 3 | 1 | 2 | 6 |
| Ma,2004 | 3 | 2 | 2 | 7 |
| Nishiura,2005 | 3 | 1 | 2 | 6 |
| Pei,2006 | 4 | 2 | 2 | 8 |
| Reynolds,2006 | 2 | 1 | 1 | 4 |
| Scales,2003 | 3 | 0 | 2 | 5 |
| Seto,2003 | 2 | 1 | 1 | 4 |
| Teleman,2004 | 3 | 2 | 2 | 7 |
| Tuan,2007 | 3 | 1 | 2 | 6 |
| Wu,2004 | 4 | 2 | 2 | 8 |
| Yin,2004 | 3 | 2 | 2 | 7 |
| Zhang, H.X.,2021 | 3 | 2 | 1 | 6 |
| Zhang, Y.,2013 | 4 | 2 | 1 | 7 |

**Supplementary Table 3.** The quality of the cohort studies

| First author, year | selection | comparability | outcome | The ratings of Newcastle-Ottawa Scale |
| --- | --- | --- | --- | --- |
| Alraddadi,2016 | 4 | 1 | 0 | 5 |
| Cheng,2010 | 3 | 2 | 2 | 7 |
| Jaeger,2011 | 3 | 2 | 2 | 7 |
| Loeb,2004 | 5 | 2 | 0 | 7 |
| Nishiyama,2008 | 2 | 1 | 2 | 5 |
| Wang, X.,2020 | 4 | 1 | 0 | 5 |
| Wang, Y.,2020 | 3 | 2 | 0 | 5 |
